# Editorial: Community series in body representation and interoceptive awareness: cognitive, affective, and social implications

**DOI:** 10.3389/fpsyg.2023.1256811

**Published:** 2023-08-17

**Authors:** Simona Raimo, Matteo Martini, Cecilia Guariglia, Gabriella Santangelo, Luigi Trojano, Liana Palermo

**Affiliations:** ^1^Department of Medical and Surgical Sciences, Magna Græcia University of Catanzaro, Catanzaro, Italy; ^2^Department of Psychology, University of East London, London, United Kingdom; ^3^Department of Psychology, “Sapienza” University of Rome, Rome, Italy; ^4^Cognitive and Motor Rehabilitation and Neuroimaging Unit, I.R.C.C.S. Santa Lucia Foundation, Rome, Italy; ^5^Department of Psychology, University of Campania “Luigi Vanvitelli”, Caserta, Italy

**Keywords:** body representation, body image, body awareness, self-perception, interoception, sense of body ownership, cardiac-perception, functional connectivity

## Introduction

The integration of multisensory signals from inside and outside the body is pivotal for building functional higher-order own body representations (BR) across the life span (Raimo et al., 2021a,b; Sorrentino et al., 2021; Boccia et al., 2023) and for self-consciousness (Park and Blanke, 2019; Quigley et al., 2021). However, exteroceptive and interoceptive bodily signals and their integrated neural representation can also influence other aspects of cognition spanning from understanding others' emotional experience (Canino et al., 2022; Raimo et al., 2023) to memory skills (Ianì, 2019; Messina et al., 2022). This point highlights the need to expand previous findings across the lifespan, providing empirical support to a specific interpretation of embodiment, according to which mental representations in bodily formats have an important role in cognitive, affective, and social processes (Goldman and de Vignemont, 2009).

Following a previous Research Topic titled “Body Representation and Interoceptive Awareness: Cognitive, Affective, and Social Implications” (Raimo et al., 2022), this Community Series features nine new articles that delve deeper into the relationship between BR, interoception, and cognitive and social processes both in childhood and adulthood.

## Interoceptive processing, self-image, and higher-order cognitive and social processes

To address the role of interoceptive ability in cognition during childhood, Pollatos et al. investigate the association between interoceptive accuracy, measured by cardiac-perception, and decision-making in a large sample of 1,454 children (6–11 years). The authors observed a correlation between interoceptive accuracy and decision-making abilities in situations of varying complexity. In line with the somatic-marker hypothesis (Damasio, 1994), these findings suggest that a better access to somatic feedback, in terms of higher interoceptive accuracy, could be positively associated with the ability to forgo short-term benefit for long-term profit already in middle childhood.

Along the same lines, Zhou et al. propose a novel model exploring how interoception promotes action understanding in children. During early infancy, infants would understand others' actions through automatic interoceptive processing and feedback. Then, in infancy, the rapid development of proprioception would provide internal reference information for imitating others' actions. Finally, in early childhood, the development of the ability to combine multiple interoceptive information would facilitate integration of different kinds of internal and external information, which is pivotal for the mentalizing level of action understanding. The involved neural mechanisms are also discussed.

In adults, sex-related individual differences in interoceptive processing are explored by Alfano et al., who investigate functional connectivity of networks involved in interoceptive sensibility in male and female participants. Behaviorally, female participants showed a stronger attitude in self-perceiving their internal sensations. An association between levels of interoceptive sensibility and functional connectivity in the salience network and in fronto-temporo-parietal brain areas is also present, more marked in female participants.

Da Costa Silva et al. validate two self-report measures of body awareness in a non-clinical adult French-speaking sample: the Postural Awareness Scale and the Multidimensional Assessment of Interoceptive Awareness (version 2). The satisfactory internal consistency, construct validity, and reliability over time suggest that they are reliable tools for assessing proprioception and interoceptive sensibility.

Kim et al. explore the relationship between mental representations of self and social evaluation, examining the usefulness of visual proxies of self-image. The authors report that a visual proxy of mental representation of self (a classification image of self) could add independent information in predicting social evaluation and suggest a new computational scoring method to objectively assess classification image of self.

Finally, Wang et al. explore the effect of transcutaneous stimulation of the vagal nerve (central in relaying visceral signals to the brain and intrinsically involved in interoception) on cognition. Vagal nerve stimulation is combined with inhibitory control training for obtaining an “exogenous” online neuromodulatory effect and an “endogenous” activation of brain regions involved in inhibitory control. The results show a synergistic ameliorative effect on inhibitory control, thus providing a novel approach to be applied in healthcare contexts.

## Higher-order functional BR and sense of body ownership

Cruz et al. present a study on the development of higher-order BR, exploring the role of body schema and body structural description (BSD) in the healthy and pathological development of children's *body image*, defined as body-related semantic-lexical knowledge (e.g., names of body parts). Using semantic word fluency tasks and graph analysis, the authors suggest that children's body image would be influenced by body schema, which is related to the sensorimotor experience, and BSD, which mainly derives from the visual experience. Also, children with cerebral palsy had poorer lexical-semantic knowledge of body parts, likely due to reduced sensorimotor and visuoperceptual experiences of the affected body parts.

Biran et al. examine BSD impairment in adults with complex regional pain syndrome (CRPS) and the consequent severe distress associated with body misrepresentation. Individuals with CRPS and healthy controls are given visual puzzles related to the human body or to non-human body objects. The participants with pain syndrome perform lower than controls only in the human body puzzle, showing a significant impairment of the topographical continuity of body parts.

Finally, Ruijia et al. focus on how individuals feel about their own deformed bodies, proposing an experimental setup called “monkey's hand”, in which the participants take a posture that creates the illusion that one has only four fingers. The results reveal an ambiguous feeling of body ownership, since participants feel that the thumb is functionally absent but structurally present. The authors suggest that this does not imply a simple lack of ownership of the thumb but implies disownership, thus proposing that disownership is different from the mere absence of ownership.

In summary, the articles included in this Community Series provide new insight on BR and interoceptive dimensions, also highlighting their importance in shaping social and cognitive processes. Despite the newfound interest in embodied perspective of higher-order cognition and the evidence collected here, a unique model of how the sensorimotor, perceptual, and interoceptive BR affect various social-cognitive activities is still lacking, highlighting the need for further research in this field.

## Author contributions

SR: Writing—original draft, Writing—review and editing. MM: Writing—review and editing. CG: Writing—review and editing. GS: Writing—review and editing. LT: Writing—review and editing. LP: Writing—original draft, Writing—review and editing.
